# Exercise Enhances Branched-Chain Amino Acid Catabolism and Decreases Cardiac Vulnerability to Myocardial Ischemic Injury

**DOI:** 10.3390/cells11101706

**Published:** 2022-05-20

**Authors:** Guiling Wu, Yanjie Guo, Min Li, Chenhan Li, Yanzhen Tan, Yueyang Li, Jia Li, Li Wang, Xing Zhang, Feng Gao

**Affiliations:** 1 Key Laboratory of Ministry of Education, School of Aerospace Medicine, Fourth Military Medical University, Xi’an 710032, China; wguiling@fmmu.edu.cn (G.W.); guoyanjie2020@fmmu.edu.cn (Y.G.); yhy2013@fmmu.edu.cn (M.L.); jiaheng@fmmu.edu.cn (C.L.); jiali816@fmmu.edu.cn (J.L.); fgao@fmmu.edu.cn (F.G.); 2 Department of Cardiovascular Surgery, Xijing Hospital, Fourth Military Medical University, Xi’an 710032, China; tianmm31@fmmu.edu.cn; 3 Department of Cardiology, Chinese PLA General Hospital, Beijing 100853, China; liyueyang@plagh.cn

**Keywords:** exercise, BCAA catabolism, myocardial infarction, PP2Cm, cardioprotection

## Abstract

Long-term exercise-induced metabolic adaptations occupy a central position in exercise-afforded cardiac benefits. Emerging evidence suggests that branched-chain amino acid (BCAA) catabolic defect contributes to cardiac dysfunction in multiple cardiometabolic diseases. However, the role of BCAA catabolism in exercise-afforded cardiac benefits remains unknown. Here, we show that exercise improves BCAA catabolism and thus reduce cardiac vulnerability to myocardial ischemic injury. Exercise increased circulating BCAA levels in both humans (male adolescent athletes) and mice (following an 8-week swimming intervention). It increased the expression of mitochondrial localized 2C-type serine-threonine protein phosphatase (PP2Cm), a key enzyme in regulating BCAA catabolism, and decreased BCAA accumulation in mouse hearts, indicating an increase in BCAA catabolism. Pharmacological promotion of BCAA catabolism protected the mouse heart against myocardial infarction (MI) induced by permanent ligation of the left descending coronary artery. Although cardiac-specific PP2Cm knockout showed no significant effects on cardiac structural and functional adaptations to exercise, it blunted the cardioprotective effects of exercise against MI. Mechanistically, exercise alleviated BCAA accumulation and subsequently inactivated the mammalian target of rapamycin in MI hearts. These results showed that exercise elevated BCAA catabolism and protected the heart against myocardial ischemic injury, reinforcing the role of exercise in the promotion of cardiac health.

## 1. Introduction

The prevalence of physical inactivity (involving about one third of adults worldwide) becomes one of the leading threats for human health nowadays [1]. In the contrary, physical activity or exercise not only reduces risks of cardiovascular disease but also alleviates cardiac vulnerability to detrimental insults [2,3,4,5,6]. The cardiovascular system is the central hub in orchestrating responses to exercise, and in turn exercise induces extensive cardiovascular adaptations, in which the changes in bioenergetics occupy a central position in exercise-afforded cardiac benefits [3,7,8].

The heart has developed a highly flexible and efficient metabolic system that can utilize almost all substrates, including carbohydrates, fatty acids, ketone bodies, lactate, and amino acids, to maintain cellular ATP homeostasis [9,10], and be capable of shifting its substrate preference in response to changes in substrate availability and other conditions. Metabolic shift plays an important role in cardiac physiological and pathological processes [11,12]. Growing evidence indicates that derangements in cardiac substrate preference contribute to the development of cardiac diseases, and reprogramming the substrate preference is a promising strategy in promoting cardiac health [11,13,14,15]. In addition to the current paradigm of lipid and glucose catabolism, emerging evidence suggests that branched-chain amino acid (BCAA) catabolism also plays a critical role in cardiac health and disease [16,17]. BCAAs, consisting of valine, leucine and isoleucine, are a group of essential amino acids, representing around 35–40% of all essential amino acids present in mammals [18]. BCAA catabolic defect is observed in numerous metabolic diseases, including obesity, diabetes, cardiac diseases, and atherosclerosis, and evidence has shown that BCAA catabolic defect plays a causal role in the development of these diseases [16,19,20]. As such, BCAA catabolic defect is one of the major metabolic hallmarks in cardiac diseases, and promoting BCAA catabolism exerts cardioprotective effects [16,17,21,22]. The major mechanism is that BCAA catabolic defect results in cardiac BCAA accumulation which disturbs cardiac growth and metabolism through activation of the mammalian target of rapamycin (mTOR) signaling [22,23].

Exercise extensively shifts substrate preference in the heart and other organs or tissues [3,4,8]. However, whether exercise regulates the utilization of BCAA in the heart remains unknown. Here, we found that exercise upregulated circulating BCAA levels and enhanced BCAA catabolism in multiple tissues. Importantly, exercise-enhanced cardiac BCAA catabolism protects the heart against myocardial ischemic injury, suggesting that the adaptation of BCAA catabolism to exercise contributes to exercise-exerted cardiac benefits.

## 2. Materials and Methods

### 2.1. Animals

All animal experiments were performed in line with the national guidelines and were approved by the Fourth Military Medical University Animal Care and Use Committee. Male wild-type C57BL/6J mice (4 weeks) were obtained from the Fourth Military Medical University Animal Center. *Ppm1k*^(flox/flox)^ C57Bl/6 mice were created by homologous recombination using an *Ppm1k* gene targeting vector with lox P sites flanking exon by Cyagen Co. (Suzhou, China). Transgenic mice expressing a non-inducible Cre recombinase transgene under the control of the myosin heavy chain 6 (Myh6) promoter/enhancer (Myh6-Cre) were purchased from Cyagen Co. (Suzhou, China). To generate cardiac-specific *Ppm1k* knockout (KO) mice (*Ppm1k*^CKO^), Myh6-Cre mice were crossed with *Ppm1k*^(flox/flox)^ mice. All mice were housed in a constant-temperature vivarium at 22 °C with a 12 h light/dark cycle.

### 2.2. Myocardial Infarction (MI)

MI was induced by permanent ligation of the left descending coronary artery as described previously [24]. Briefly, mice were anesthetized with 1% pentobarbital sodium (0.1 g/kg), and the left descending coronary artery near its origin from the left coronary artery was ligated using 4–0 silk suture. Sham-operated mice underwent the same surgery but without ligation. The area at risk was stained with Evans blue after ligation of the left descending coronary artery (Figure S1). Mice were randomized to receive vehicle (0.5 mL saline), BCAA, or 3,6-dichlorobenzo (β) thiophene-2-carboxylic acid (BT2, an activator of BCKD) (MecChemExpress, Monmouth Junction, NJ, USA, #HY-114855) treatment after surgery. Mice were treated with BCAA mixture (weight ratio, leucine:valine:isoleucine = 2:1:1; Sigma-Aldrich, St. Louis, MO, USA, #L8912, #V0513, #I2752) (1.5 mg/g/day solved in 0.5 mL saline) or BT2 (40 mg/kg/day diluted in 0.5 mL saline) by oral gavage for 4 weeks.

### 2.3. Plasma Metabolomics and Bioinformatic Analysis

Blood samples were collected from a human study reported previously [25]. All human studies were approved by the Human Research Ethics Committee of Fourth Military Medical University. A total of 32 healthy young (aged 19–22 years) men were studied: 16 student athletes and 16 untrained control students. Please refer to the previous report for detailed information [25]. Metabolomic profiling was conducted by liquid chromatography-tandem mass spectrometry on the UHPLC system (Agilent Technologies, Santa Clara, CA, USA, #1290) by Gene Denovo Biotechnology Co. (Guangzhou, China). After data preprocessing and annotation, multivariate statistical analysis was performed. Orthogonal projection to latent structures-discriminant analysis (OPLS-DA) was used in comparison groups with R package models. A variable importance in projection (VIP) score was used to rank the metabolites distinguished between two groups. *t*-test was used for screening differential metabolites. Those with a *p* value < 0.05 and VIP ≥ 1 were considered differential metabolites. Differential metabolites were then mapped to KEGG metabolic pathways for pathway analysis and enrichment analysis.

### 2.4. Neonatal Rat Cardiomyocyte Isolation and Culture

Neonatal rat cardiomyocytes were isolated from new-born SD rats as previously reported [11]. Briefly, the left ventricles were harvested, minced and digested with collagenase Ⅱ (Biofrox, Hessen, Germany, #9001-12-1). Cells were cultured in Dulbecco’s Modified Eagle Medium-F12 with 10% fetal bovine serum (BI Co., Kibbutz Beit Haemek, Israel, #04-001-1ACS) and 1% penicillin-streptomycin (HyClone Co., Logan, UT, USA, #SV30010) at 37 °C in 5% CO_2_. Hypoxia (12 h) was induced by culturing the cells in serum- and glucose-free medium in an air-tight Plexiglas chamber as previously described [26]. Normoxic cells were cultured without intervention. Cells were treated with BCAA mixture (weight ratio, leucine:valine:isoleucine = 2:1:1; Sigma-Aldrich, St. Louis, MO, USA) with a concentration of 3 mM at 10 min before hypoxia, and BCAA mixture were maintained in the culture medium during hypoxia. Recombinant adenovirus-*Ppm1k* (Ad-*Ppm1k*) and the negative control adenovirus (Ad-NC) were constructed and packaged (Han Bio Co., Shanghai, China), and the titer of adenovirus is 1 × 10^9^. Once the cells reached 70% confluence, they were infected with Ad-Ppm1k or Ad-NC (2 μL/mL). Cells were exposed to normoxia or hypoxia at 60 h post infection.

### 2.5. Exercise Protocol

Swimming exercise was conducted as previously described [25]. Mice (4 weeks) swam 90 min once daily for 5 days a week for 8 weeks. Mice were adapted to swimming training with a 10 min session on the first day. Sessions were then progressively increased to 90 min/day over a 1-week period. All exercise sessions took place during 10:00 p.m.–12:00 p.m. Sedentary mice were age-matched and were kept in the same rearing condition without swimming. The mice (12 weeks) were subjected to sham or MI surgery at one day after exercise.

### 2.6. BCAA Concentration Analysis

BCAA concentration was determined with a commercially available BCAA detection kit (Biovision, Milpitas, CA, USA, #K564-100) following the manufacturer’s instructions. The absorbance value of each sample was measured at 450 nm in a microplate reader and calculated based on the standard curve.

### 2.7. BCKD Enzyme Activity

BCKD activity was assessed as described previously [27]. Briefly, BCKD complex was isolated from fresh tissue extracts with 9% polyethylene glycol, and BCKD enzyme activity was determined spectrophotometrically by measuring the rate of NADH production resulting from the conversion of α-ketoisovalerate into isobutyryl-CoA. NADH content was recorded by the absorbance at 340 nm.

### 2.8. Evaluation of Cardiac Function

Mice were anesthetized by 2% isoflurane and ventricular function was determined by an echocardiographic imaging system (Vevo 2100, VisualSonics, Toronto, ON, Canada) at 4 weeks after sham or MI operation. Two-dimensional echocardiographic views of the mid-ventricular short axis were collected at the section of the papillary muscle tips below the mitral valve. The left ventricular ejection fraction (LVEF) and end-diastolic interventricular septal thickness (IVS; d) were calculated. For hemodynamic examination, mice were anesthetized by 2% isoflurane and a 1.4 F micromanometer-tip catheter (Miller Inc., Huntsville, AL, USA, #SPR839) was intubated into the left ventricle to determine the left ventricular pressure. The instantaneous first derivation of the left ventricular pressure (+dP/dt_max_ and −dP/dt_max_) was calculated.

### 2.9. Evaluation of Apoptosis

To evaluate apoptosis, TdT-mediated dUTP nick-end labeling (TUNEL) staining (Servicebio, China, #GDP1042) was performed in cardiac sections and cultured cells following the manufacturer’s instructions. TUNEL/DAPI double-positive nuclei were counted as apoptotic cells.

### 2.10. Histological Analysis

Fresh cardiac tissues were fixed with 4% paraformaldehyde for more than 24 h. Tissues were then embedded and sectioned at a thickness of 5 μm. The slices were then dewaxed with standard protocols. Slices were stained with hematoxylin and eosin (Sigma-Aldrich, St. Louis, MO, USA) following the manufacturer’s instructions. Masson staining and wheat germ agglutinin (WGA) immunofluorescence were performed as described previously [15]. For measuring the capillary density, CD31 immunofluorescence was conducted as described previously [4]. WGA or CD31 fluorescence was acquired with an inverted confocal microscope (Zeiss LSM 800). ImageJ software (1.53a, NIH, Bethesda, MD, USA) was used to analyze the images. CD31 images in the ischemic territory near the non-infarct area were shown and analyzed.

### 2.11. Assessment of Exercise Ability

For measurement of forelimb strength, a grip strength meter (BIO-GS3, Bioseb, FL, USA) was used. Forelimbs of mice were put on the grid and free to grip the grid. Then, mice tails were gently pulled back and their torsos were kept parallel with the grid simultaneously to eliminate the interference of the back strength. Forelimb grip strength of each mouse was measured 5 times discontinuously and calculated the average value. Endurance exercise ability was measured as previously described [28]. Briefly, mice were acclimated to the treadmill (ZH-PT/5S Huaibei Zhenghua Biologic Apparatus Facilities Ltd. Co., Anhui, China) for 3 consecutive days with a speed of 15 m/min lasting 20 min each day prior to measurement. For endurance testing, the speed started at 13 m/min (5° inclination) for 5 min, and was then increased by 1 m/min every min until 20 m/min and kept constant for 20 min. Every 20 min, the speed was increased by 5 m/min. Mice were considered exhausted when they sat on the shocker plate for more than 10 s without attempting to reinitiate running.

### 2.12. Western Blot

Proteins from tissues and cells were isolated using radioimmunoprecipitation assay lysis buffer (Beyotime, Shanghai, China, #P0013C) containing protease inhibitors cocktail (Roche#11836170001) and phosphatase inhibitors (Sigma-Aldrich, St. Louis, MO, USA, #4906845001). The protein concentration was measured with a bicinchoninic acid protein assay kit (Beyotime, Shanghai, China, #P0012). The protein expression and phosphorylation were measured as described previously [29]. The primary antibodies were as follows: p-BCKDHA (1:1000, Abcam, Cambridge, MA, USA, #ab200577), BCKDHA (1:1000, Abcam, #ab126173), PP2Cm (1:1000, Abcam, #ab135286), BCKDK (1:1000, Abcam, #151297), p-mTOR (1:1000, CST, Danvers, MA, USA, #2971S), mTOR (1:1000, CST, #2972S), p-ribosomal S6 kinase (p-S6K, 1:1000, CST, #9204S), S6K (1:1000, CST, #2708S), cleaved caspase 3 (1:1000, CST, #9661S), caspase 3 (1:1000, CST, #9962S) and α-Tubulin (1:1000, CST, #2125).

### 2.13. Statistical Evaluation

All values are presented as mean ± SEM. The results were compared with one-way analysis of variance or two-way analysis of variance, with all tests followed by an unpaired, two-tailed *t* test, as appropriate. Bonferroni’s correction for multiple comparisons was applied. *p* < 0.05 was considered to be significant difference.

## 3. Results

### 3.1. Exercise Elevated Circulating BCAA Levels in Humans

**Figure 1:**
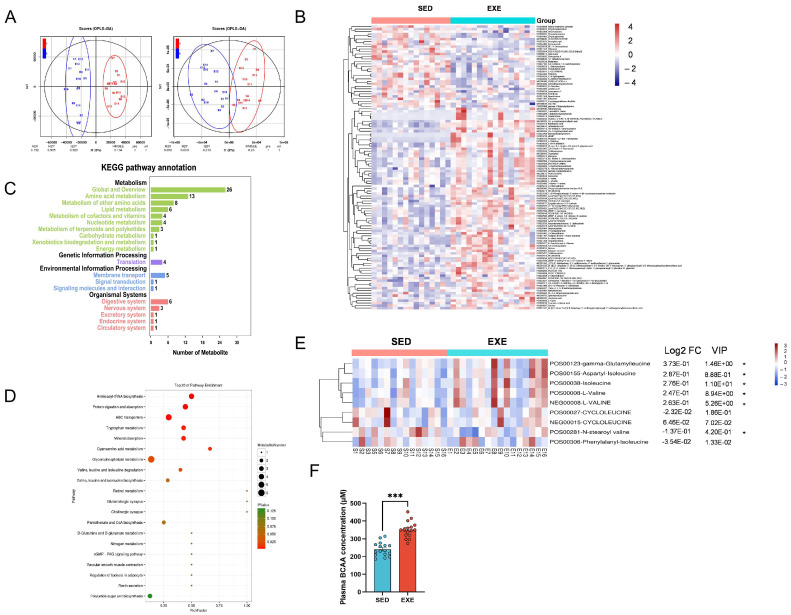
Exercise upregulated circulating BCAA levels in humans. (**A**). The clustering of orthogonal projection to latent structures-discriminant analysis (OPLS-DA) in positive (left panel) and negative (right panel) ion mode. (**B**). Heat map of the differentially concentrated metabolites in plasma from trained and untrained subjects. (**C**). The KEGG pathways involved in the differentially concentrated metabolites. (**D**). Pathway enrichment analysis indicated that both BCAA degradation and biosynthesis were changed obviously. (**E**). Heat map of all detected BCAAs. (**F**). Circulating BCAA concentrations in trained and untrained subjects. Values are presented as mean ± SEM. *n* = 16 subjects for each group. * *p* < 0.05; *** *p* < 0.001.

We recruited a total of 32 healthy young men aged 19–22 years, including 16 student athletes and 16 untrained control students [25]. Non-targeted metabolomics profiling of plasma samples was conducted to identify the metabolic modulation of exercise. A total of 3019 named metabolites comprising all major metabolic groups were detected. Blood samples from untrained and trained students showed differential metabolite profiles (Figure 1A). A total of 114 differentially concentrated metabolites (*p* < 0.05 and VIP ≥ 1) were identified, among which 81 metabolites were upregulated and 33 metabolites were downregulated (Figure 1B, Table S1). Bioinformatic analysis revealed that amino acid metabolism pathway was the most significant changed pathway between blood samples from untrained and trained students (Figure 1C,D). Pathway enrichment analysis indicated that both BCAA degradation and biosynthesis were changed obviously (Figure 1D), suggesting BCAA metabolism may play an important role in exercise biology. Among the detected BCAAs, 4 BCAAs (44%) were increased and others were unchanged (Figure 1E). In fact, quantitative detection of BCAA revealed that circulating BCAA concentrations were upregulated in trained students (Figure 1F).

### 3.2. Exercise Enhanced Cardiac BCAA Catabolism in Mice

**Figure 2:**
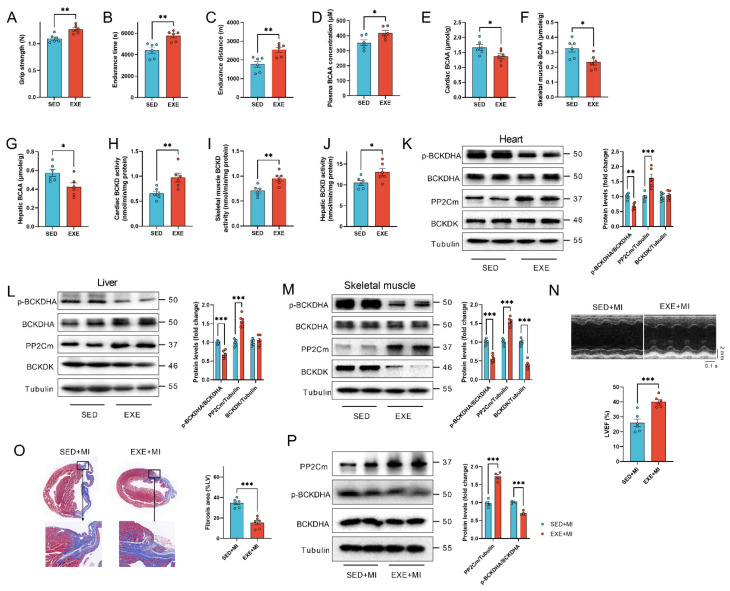
Exercise enhanced cardiac BCAA catabolism in mice. (**A**–**C**). Exercise increased grip strength (**A**), and treadmill performance (**B**,**C**) in mice. (**D**). Exercise increased circulating BCAA levels in mice. (**E**–**G**). Exercise decreased BCAA contents in hearts (**E**), skeletal muscles (**F**) and livers (**G**). (**H**–**J**). Exercise increased the activity of BCKD in hearts (**H**), skeletal muscles (**I**) and livers (**J**). (**K**). Exercise increased PP2Cm contents and decreased the phosphorylation levels of BCKDHA in hearts. (**L**). Exercise increased PP2Cm contents and decreased the phosphorylation levels of BCKDHA in livers. (**M**). Exercise increased PP2Cm contents, and decreased BCKDHA phosphorylation and BCKDK expression in skeletal muscles. (**N**). Exercise preconditioning increased the left ventricular ejection fraction (LVEF) in MI hearts at 4 weeks post-surgery. (**O**). Exercise preconditioning decreased cardiac fibrosis in MI hearts at 4 weeks post-surgery. The blue staining indicates collagen and fibrosis. (**P**). Exercise preconditioning increased cardiac PP2Cm content and decreased BCDHA phosphorylation in MI hearts at 4 weeks post-surgery (*n* = 4). Values are presented as mean ± SEM. *n* = 6. * *p* < 0.05; ** *p* < 0.01; *** *p* < 0.001.

An 8-week swimming intervention exercise increased the grip strength and treadmill performance in mice (Figure 2A–C). It also increased the circulating BCAA levels in mice (Figure 2D), but decreased the accumulation of BCAA in hearts, skeletal muscles and livers (Figure 2E–G). Branched-chain α-ketoacid dehydrogenase (BCKD) complex in mitochondria is the rate-limiting enzyme for BCAA catabolism. BCKD can be phosphorylated and inactivated by BCKD kinase (BCKDK), or dephosphorylated and activated by a mitochondrial localized 2C-type serine-threonine protein phosphatase (PP2Cm) [22,23]. Exercise increased the activity of BCKD by 48.4%, 35.7%, and 24.6% in hearts, skeletal muscles and livers, respectively (Figure 2H,J), and decreased the phosphorylation of BCKD E1 subunit alpha (BCKDHA), a component of BCKD complex, in these tissues (Figure 2K–M). It also increased the expression of PP2Cm in hearts, skeletal muscles and livers (Figure 2K–M). Exercise decreased BCKDK expression in skeletal muscles but not in hearts and livers (Figure 2K–M). These results suggested that exercise increases BCAA catabolism, and decreases the BCAA accumulation in multiple tissues. These mice were subjected to MI after exercise. Exercise preconditioning protected the heart against MI, as evidenced by increased LVEF and decreased cardiac fibrosis at 4 weeks post-surgery (Figure 2N,O). In addition, exercise preconditioning increased cardiac PP2Cm content and decreased BCDHA phosphorylation in MI hearts at 4 weeks post-surgery (Figure 2P).

### 3.3. Enhancement of BCAA Catabolism Protected the Heart against MI

**Figure 3:**
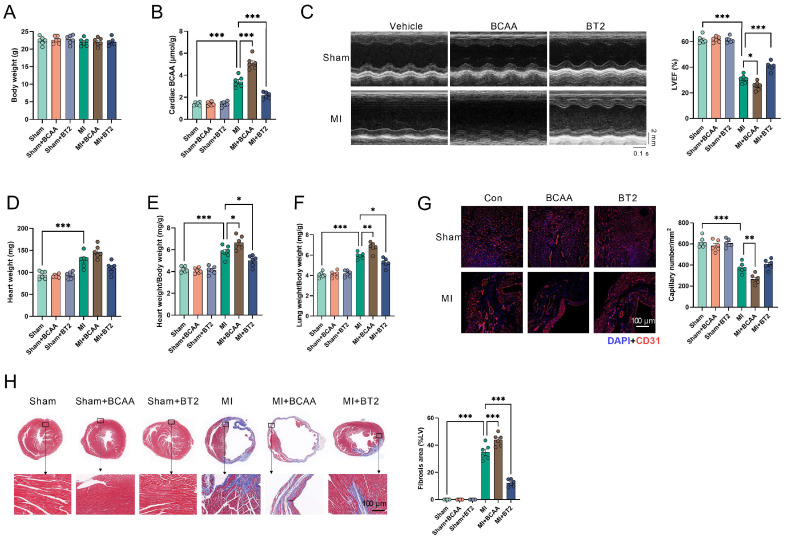
Enhancement of BCAA catabolism protected the heart against MI. Mice were subjected to sham or MI operation and treated with either BCAA or BT2 post-surgery for 4 weeks. All parameters were detected at 4 weeks post-surgery. (**A**). Body weight of mice. (**B**). Cardiac BCAA levels in sham and MI mice. (**C**). Enhancement of BCAA catabolism by BT2 increased the left ventricular ejection fraction (LVEF) in mice with MI. (**D**). Heart weight in sham and MI mice. (**E**). Heart weight–body weight ratio in sham and MI mice. (**F**). Lung weight–body weight ratio in sham and MI mice. (**G**). Cardiac capillary density in the ischemic territory near the non-infarct area as detected by CD31 fluorescence in sham and MI mice. (**H**). Enhancement of BCAA catabolism by BT2 decreased cardiac fibrosis as detected by Masson staining in mice with MI. The blue staining indicates collagen and fibrosis. Values are presented as mean ± SEM. *n* = 6. * *p* < 0.05; ** *p* < 0.01; *** *p* < 0.001.

Next, mice were subjected to MI and treated with either BCAA or BT2, an activator of BCKD (post-surgery for 4 weeks), to test whether circulating BCAA or BCAA catabolism exerts cardioprotective effects. The body weight and cardiac function were comparable between control sham mice and sham mice treated with BCAA or BT2 (Figure 3A–F). MI induced cardiac BCAA accumulation, and BCAA treatment aggravated BCAA accumulation in MI mice (Figure 3B). Supplementation of BCAA aggravated cardiac function as evidenced by decreased LVEF in mice with MI (Figure 3C). Although BCAA supplementation showed no significant effects on heart weight, it increased the heart weight–body weight ratio and the lung weight–body weight ratio in mice with MI (Figure 3D–F). In addition, BCAA supplementation aggravated cardiac fibrosis and decreased cardiac capillary density in mice with MI (Figure 3G,H), suggesting that BCAA supplementation aggravated cardiac injury in mice with MI. In contrast, BT2 treatment decreased cardiac BCAA content and improved cardiac function as evidenced by increased LVEF in mice with MI (Figure 3B,C). It decreased the heart weight–body weight ratio and the lung weight–body weight ratio (Figure 3D–F), and alleviated cardiac fibrosis in mice with MI (Figure 3H), suggesting a cardioprotective effect of BT2. These results suggested that enhancement of BCAA catabolism, but not increasing circulating BCAA levels, exerts cardioprotective effects against MI.

### 3.4. Cardiac-Specific PP2Cm KO Did Not Affect Exercise-Induced Cardiac Remodeling

**Figure 4:**
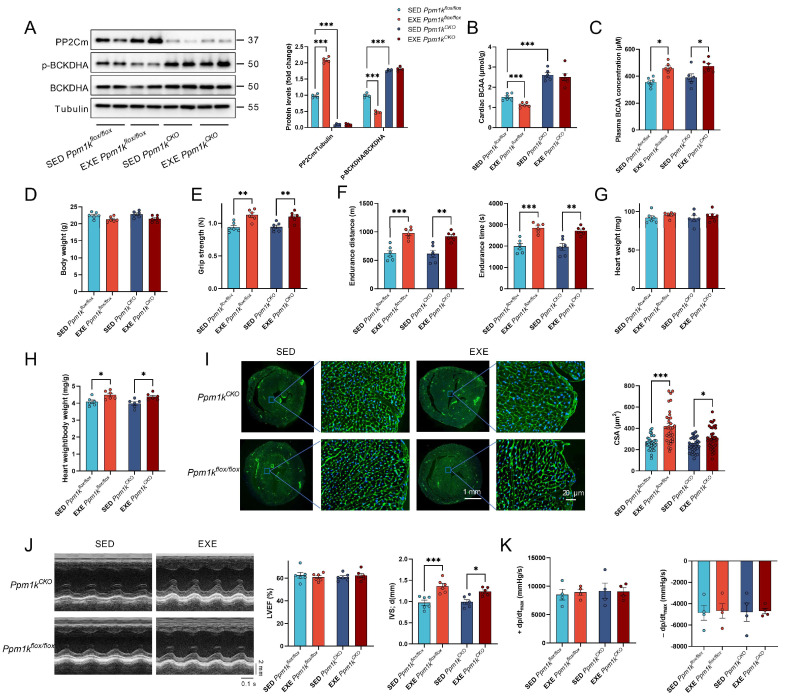
Cardiac-specific PP2Cm KO showed little effects on cardiac remodeling to exercise. (**A**). Exercise increased the expression of PP2Cm and decreased the phosphorylation of BCKDHA in the heart from control (*Ppm1k*^flox/flox^) mice but not KO (*Ppm1k*^CKO^) mice. (**B**). Cardiac BCAA content in mice subjected to sedentary or exercise in control and KO mice. (**C**). Plasma BCAA concentrations in mice subjected to sedentary or exercise in control and KO mice. (**D**). Body weight of mice subjected to sedentary or exercise in control and KO mice. (**E**,**F**). Exercise increased grip strength (**E**), and treadmill performance (**F**) in both control and KO mice. (**G**). Heart weight of mice subjected to sedentary or exercise in control and KO mice. (**H**). Exercise increased the heart weight–body weight ratio in both control and KO mice. (**I**). Exercise-induced cardiac hypertrophy in both control and KO mice. Typical images of cardiac WGA staining were shown in left. Green, WGA; blue, DAPI. The quantified results of the cross-sectional area (CSA) of cardiomyocytes were shown in right. (**J**). Cardiac systolic function in mice subjected to sedentary or exercise in control and KO mice. Typical images of cardiac systolic function as detected by echocardiography were shown in left, and quantified results of ventricular ejection fraction (LVEF) and end-diastolic interventricular septal thickness (IVS; d) were shown in right. (**K**). ±dP/dt_max_ in mice subjected to sedentary or exercise in control and KO mice (*n* = 4). Values are presented as mean ± SEM. *n* = 6. * *p* < 0.05; ** *p* < 0.01; *** *p* < 0.001.

It has been shown that PP2Cm is a key enzyme in the regulation of BCAA catabolism. To examine the role of BCAA in cardiac adaptation to exercise, a cardiac-specific PP2Cm KO model was established. Control (*Ppm1k*^flox/flox^) and KO (*Ppm1k*^CKO^) mice were subjected to an 8-week exercise intervention or remained sedentary. Exercise increased the expression of PP2Cm and decreased the phosphorylation of BCKDHA in the heart from control mice but not KO mice (Figure 4A). It also decreased the cardiac BCAA levels in control mice but not KO mice (Figure 4B). Exercise increased the circulating BCAA levels in both control and KO mice (Figure 4C). There are no differences in body weight, physical capacity (as detected by the grip strength and treadmill performance), and cardiac structure and function between control sedentary and KO sedentary mice (Figure 4D–J), suggesting that cardiac-specific PP2Cm KO displayed little effects on overall and cardiac health. Exercise showed no significant effects on body weight, but increased grip strength, and treadmill performance in both control and KO mice (Figure 4D–F). In addition, exercise showed no significant effects on heart weight, and cardiac systolic function as assessed by LVEF, but increased the heart weight–body weight ratio, the cross-sectional area of cardiomyocytes, and the end-diastolic interventricular septal thickness in both control and KO mice (Figure 4G–J). The hemodynamic examination also confirmed that exercise showed no significant effects on cardiac function in both control and KO mice (Figure 4K). These results indicated that exercise-induced PP2Cm upregulation contributes little to cardiac structural adaptations to exercise.

### 3.5. Cardiac-Specific PP2Cm KO Blunted Exercise-Induced Cardioprotective Effects against MI

**Figure 5:**
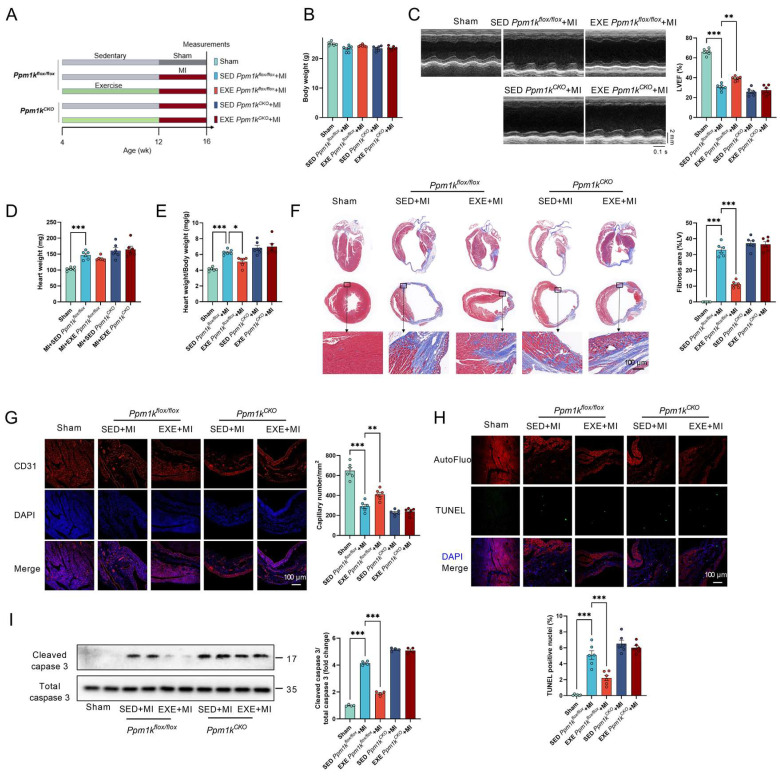
Cardiac-specific PP2Cm KO abolished the cardioprotective effects of exercise. (**A**). The control (*Ppm1k*^flox/flox^) and KO (*Ppm1k*^CKO^) mice were subjected to MI after an 8-week exercise intervention to test whether PP2Cm upregulation contributes to exercise-induced cardioprotection. (**B**). Exercise preconditioning showed no significant effects on body weight in both control and KO mice with MI. (**C**). Exercise preconditioning increased cardiac systolic function as detected by echocardiography in control but not KO mice with MI. (**D**,**E**). Heart weight (**D**) and heart weight–body weight ratio (**E**) in mice subjected to MI in control and KO mice. (**F**). Cardiac structure and fibrosis as detected by Masson staining in mice subjected to MI in control and KO mice. The blue staining indicates collagen and fibrosis. (**G**). Exercise preconditioning increased cardiac capillary density (the ischemic territory near the non-infarct area) as detected by CD31 immunofluorescence in control but not KO mice with MI. (**H**). Exercise preconditioning attenuated cardiac apoptosis as detected by TUNEL staining in control but not KO mice with MI. (**I**). Caspase 3 and cleaved caspase 3 contents in control and KO mice (*n* = 4). Values are presented as mean ± SEM. *n* = 6. * *p* < 0.05; ** *p* < 0.01; *** *p* < 0.001.

The control and KO mice were subjected to MI after an 8-week sedentary/exercise intervention to test whether PP2Cm upregulation contributes to exercise-induced cardioprotection (Figure 5A). Exercise preconditioning showed no significant effects on body weight in both control and KO mice with MI (Figure 5B). Exercise preconditioning increased the LVEF value by 28.5% in control but not KO mice with MI (Figure 5C). It also decreased the heart weight–body weight ratio in control but not KO mice with MI (Figure 5D,E). In addition, exercise preconditioning alleviated cardiac hypertrophy, decreased cardiac fibrosis, increased cardiac capillary density, and decreased cardiac apoptosis and caspase 3 cleavage in control mice with MI, while these protective effects exerted by exercise preconditioning were abolished in cardiac-specific PP2Cm KO mice (Figure 5F–I). These results suggested that exercise preconditioning protects the heart against MI through upregulation of PP2Cm.

### 3.6. Exercise Preconditioning Reduced Myocardial BCAA Accumulation and the Resultant mTOR Activation in MI Hearts

**Figure 6:**
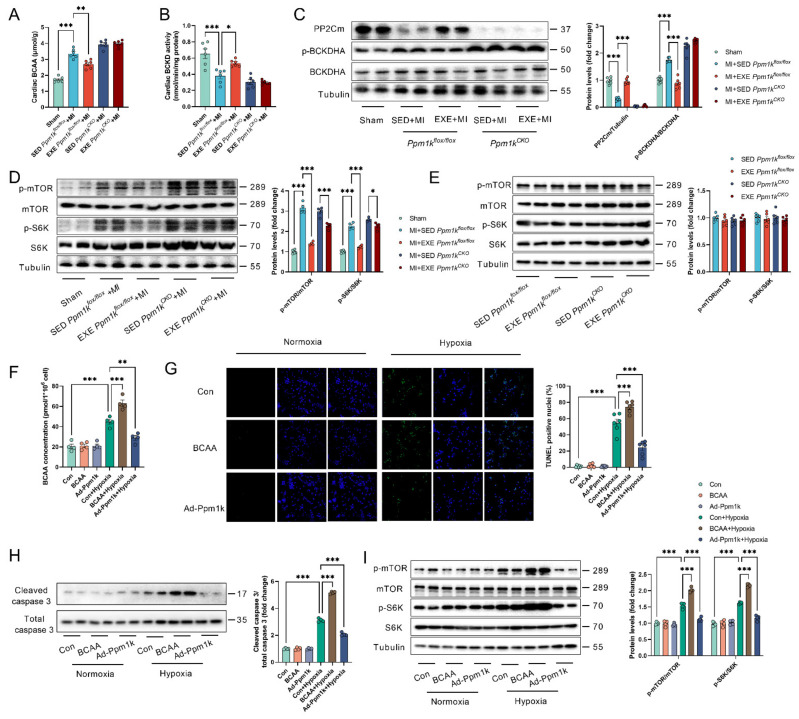
Exercise preconditioning alleviated BCAA accumulation and the resultant mTOR activation in hearts with MI. (**A**). Exercise preconditioning decreased BCAA accumulation in MI hearts from control (*Ppm1k*^flox/flox^) but not KO (*Ppm1k*^CKO^) mice. (**B**). Exercise preconditioning increased BCKD activity in MI hearts from control but not KO mice. (**C**). Exercise preconditioning increased PP2Cm content and decreased BCKDHA phosphorylation in MI hearts from control mice but not PP2Cm KO mice. (**D**). Exercise preconditioning alleviated the activation of mTOR signaling in MI hearts from control but not PP2Cm KO mice. (**E**). Exercise showed no significant effects on cardiac mTOR signaling in both control and PP2Cm KO mice. (**F**). PP2Cm overexpression alleviated intracellular BCAA accumulation in cardiomyocytes under hypoxia (*n* = 4). (**G**). PP2Cm overexpression alleviated cardiomyocyte apoptosis under hypoxia. (**H**). PP2Cm overexpression decreased caspase 3 cleavage in cardiomyocytes under hypoxia (*n* = 4). (**I**). PP2Cm overexpression alleviated mTOR activation, while BCAA treatment aggravated mTOR activation in cultured cardiomyocytes under hypoxia. Values are presented as mean ± SEM. *n* = 4 in (**D**,**F**,**H**,**I**), and *n* = 6 in other panels. * *p* < 0.05; ** *p* < 0.01; *** *p* < 0.001.

Evidence has shown that BCAA accumulation and the resultant mTOR activation underlies BCAA catabolic defect-induced adverse effect in MI [24,30]. Thus, we next examined the BCAA accumulation and mTOR signaling in MI hearts. MI induced cardiac BCAA accumulation and BCAA catabolic defect as evidenced by decreased BCKD activity, downregulated PP2Cm content and increased BCKDHA phosphorylation in hearts (Figure 6A–C). Exercise preconditioning improved BCAA catabolism and decreased BCAA accumulation in MI hearts from control but not KO mice (Figure 6A–C). Consistently, MI increased mTOR activation as evidenced by increased phosphorylation of mTOR and S6K, an effector of mTOR signaling, and exercise preconditioning decreased mTOR activation in MI hearts from control but not KO mice (Figure 6D). In addition, exercise alone showed no significant effects on cardiac mTOR activation in both control and KO mice (Figure 6E). These results reinforced the notion that exercise preconditioning protects the heart against MI through improving BCAA catabolism. We also tested the effects of PP2Cm overexpression in cultured neonatal cardiomyocytes under normoxia and hypoxia. BCAA supplementation showed no significant effects on mTOR activation and cell apoptosis in cultured cardiomyocytes under normoxia, but increased intracellular BCAA accumulation, aggravated cell apoptosis, increased caspase 3 cleavage, and aggravated mTOR activation in cardiomyocytes under hypoxia (Figure 6F–I). In contrast, PP2Cm overexpression alleviated mTOR activation and cell apoptosis in cardiomyocytes under hypoxia (Figure 6F–I).

## 4. Discussion

The metabolic adaptations to exercise occupy a central position underlying the mechanisms of exercise-induced cardioprotection. In the present study, we found that exercise upregulated circulating BCAA levels in both humans and mice, and increased BCAA catabolism in multiple organs or tissues, including the heart. Importantly, the upregulation of BCAA catabolism in the heart contributes to exercise-exerted cardioprotection through alleviation of cardiac BCAA accumulation in pathological condition. These findings reveal a novel mechanism of exercise-afforded cardiac benefits that are mediated by promotion of BCAA catabolism.

BCAAs not only serve as substrates for protein synthesis, but also participate in energy homeostasis and signal transduction. Circulating BCAA homeostasis is governed by protein turnover, BCAA intake, and BCAA catabolism. Although BCAA supplementation has been thought to promote anabolic pathways and therefore mitigate cachexia, attenuate fatigue during exercise, promote wound healing, and stimulate insulin production, increased circulating BCAA concentrations are associated with the pathogenesis of various metabolic diseases, including obesity and diabetes [19,31,32,33,34]. Here, we observed that long-term exercise elevated circulating BCAA levels in humans and mice. However, different from metabolic diseases, exercise-induced elevation in circulating BCAA is not associated with metabolic disorder and other adverse effects. The difference lies in that BCAA catabolism is enhanced by exercise, while it is impaired in metabolic diseases [35]. It is well established that both acute and long-term exercise increases BCAA oxidation in skeletal muscles and livers [36,37,38]. Thus, the capacity of BCAA catabolism, rather than circulating BCAA concentrations, determines the biological significance of BCAAs. Exercise exerts profound effects on protein turnover. In general, exercise increases autophagy and other protein degradation-related processes, which may contribute to the elevated circulating BCAA levels induced by exercise [39,40,41].

The capacity of BCAA catabolism is also a determinant in the maintenance of intracellular BCAA homeostasis [16,17,22,23,24]. Although BCAA catabolism contributes less to cardiac biogenetics, the enzymes in BCAA catabolic pathway are highly expressed in the heart [42]. The capacity of BCAA catabolism is impaired in cardiac diseases, accompanied with the downregulation of PP2Cm, leading to cardiac BCAA accumulation [16,17,22,23,24]. Recent studies highlight the role of BCAAs as nutrient signal molecules in regulating cellular metabolism and growth. BCAA accumulation induces oxidative stress and metabolic disorder, which contributes to the pathogenesis of cardiac diseases [43,44]. Thus, supplementation of BCAA aggravated cardiac dysfunction, while improving BCAA catabolism, exerted cardioprotective effects against MI. Exercise upregulated PP2Cm expression and BCAA catabolism in multiple tissues, while cardiac-specific PP2Cm KO abolished the cardioprotective effects of exercise, suggesting the critical role of BCAA catabolic adaptation in exercise-exerted cardiac benefits. Evidence has shown that activation of mTOR signaling plays an indispensable role in BCAA accumulation-induced adverse effects on cardiac function [24,30]. Our results showed that the level of mTOR activation was consistent with intracellular BCAA contents and cardiac function in hearts with MI, reinforcing the critical role of BCAA catabolism and mTOR signaling in the pathogenesis of MI. It should be noted that BCAA accumulation and mTOR activation were not observed in sham mice and normoxic cells even when they were treated with BCAA.

Interestingly, cardiac PP2Cm KO showed no significant effects on the cardiac function in mice without MI, consistent with a previous report [16]. The cardiac adaptations to exercise, namely the physiological hypertrophy, were comparable between control and KO mice, suggesting that other compensatory mechanisms are capable of maintaining cardiac BCAA homeostasis and PP2Cm is not critical in the maintenance of cardiac function in physiological conditions. In addition, the increases in exercise capacity in response to exercise as detected by the grip strength and treadmill performance were comparable between control and KO mice, suggesting that cardiac PP2Cm contributes little to physical performance in response to exercise. Of note, cardiac-specific PP2Cm KO abolished the exercise preconditioning-induced cardioprotection against MI, suggesting that exercise-upregulated BCAA catabolism plays an important role in pathological conditions.

Taken together, exercise enhances cardiac BCAA catabolism through the upregulation of PP2Cm in the heart, which alleviates the cardiac vulnerability to myocardial ischemic injury. These findings reveal that exercise increases cardiac resistance to detrimental insults through elevating BCAA catabolism, reinforcing the role of exercise in the promotion of cardiac health.

## 5. Limitations

Although it is well established that exercise exerts extensive beneficial effects on human health, the clinical implications of exercise in the prevention and treatment of cardiovascular disease still present a challenge [45,46,47,48,49]. The FITT (frequency, intensity, time, and type) principle of exercise is a framework for exercise prescription in the clinic. Here, we only showed that long-term aerobic exercise promotes BCAA metabolism and exerts protective effects against MI in mice. The effects of a different intensity, frequency, and type of exercise on cardiac BCAA metabolism remain unknown. The other limitation of the study is that exercise exerts beneficial effects through multiple mechanisms and the exact contribution of cardiac BCAA catabolism to exercise-induced cardioprotection needs to be examined. In addition, there are some issues which were raised by the reviewers, especially in the methods. Firstly, neonatal mouse cardiomyocytes were suggested to be used instead of neonatal rat cardiomyocytes in this study. Secondly, we failed to detect an increase in circulating BCAA levels in response to exercise in long-term frozen samples from Ppm1k^flox/flox^ mice, possibly due to the metabolite degradation, suggesting that fresh samples should be used to detect the metabolites in studying BCAA. Finally, the hemodynamic data should be provided in all experiments in evaluating cardiac function.

## Data Availability

The datasets used and/or analyzed during this study are available from the corresponding author on reasonable request.

## Supplementary Materials

The following supporting information can be downloaded at: https://www.mdpi.com/article/10.3390/cells11101706/s1, Table S1: Differentially concentrated metabolites; Figure S1: Cardiac area at risk stained with Evans blue after ligation of left descending coronary artery in mice.
