# Supplementary material for: Exercise Enhances Branched-Chain Amino Acid Catabolism and Decreases Cardiac Vulnerability to Myocardial Ischemic Injury
Source: Cells. 2022 May 20;11(10):1706. doi: 10.3390/cells11101706 (PMC9139679; doi:10.3390/cells11101706)
Supplement: Supplementary file 1 [file cells-11-01706-s001.zip › cells-1570635-supplementary.pdf]

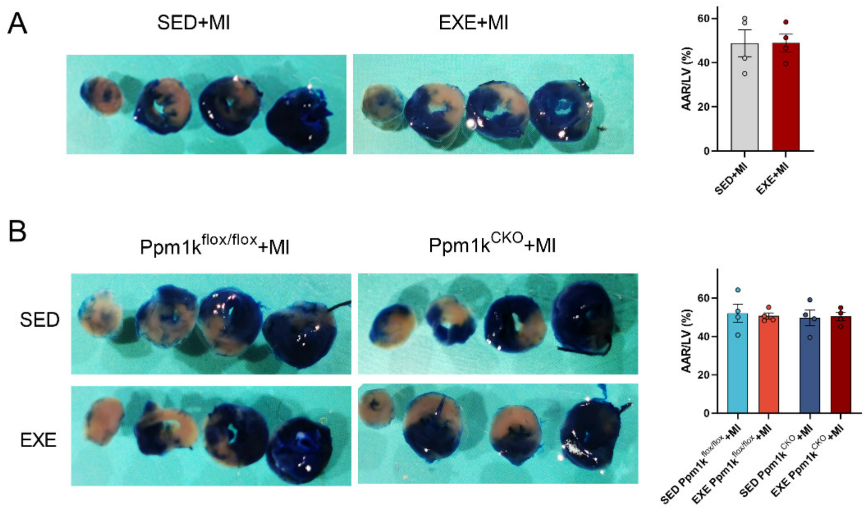

Figure S1. Myocardial area at risk after MI. A. Representative photographs of LV sections and myocardial area at risk as a percentage of LV in sedentary and exercise group after MI. B. Representative photographs of LV sections and myocardial area at risk as a percentage of LV in sedentary Ppm1k$^{flox/flox}$ and Ppm1k^{CKO} and exercise Ppm1k$^{flox/flox}$ and Ppm1k^{CKO} group. Blue portion, non-ischemic normal region; red portion, myocardial area at risk.

Table S1. Differentially concentrated metabolites.

Name	log2_FC(case mean/control mean)	P value	VIP
dUMP	4.278826	0.016664	1.143099
DODEC-2-ENE-8,10-DIYNOIC ACID ISOBUTYLAMIDE	4.152134	0.041907	6.651161
Monobromobisphenol A	4.101963	0.016384	2.741813
Fawcettimine	4.063611	0.042633	8.461916
Tulobuterol hydrochloride	3.435245	0.038239	3.515647
L-Histidine	2.501883	6.27E-05	9.16746
Propane-1,2-diol 1-phosphate	2.172405	0.000311	2.093217
Tramadol N-Oxide	1.747885	0.049128	1.472309
Se-Methyl-L-selenocysteine	1.469562	0.001786	2.639209
DL-p-Hydroxyphenyllactic acid	1.015997	5.41E-07	2.480248
Gentisinic acid	0.842578	0.038154	1.348337
15-15-methyl PGF2 ethyl amide	0.835755	0.000168	1.282084
Nitrogen mustard	0.803172	7.78E-05	1.599091
Metobromuron	0.736506	0.009958	1.233196
Indolelactic acid	0.720804	3.54E-07	1.607432
Indolelactic acid	0.711064	1.93E-07	1.747982
DL-3,4-Dihydroxymandelic acid	0.684959	0.027915	2.047504
Nitrofurantoin	0.642433	0.040237	1.008505
N-oleoyl taurine	0.587157	0.001671	1.05289
Trans-2, 3, 4-Trimethoxycinnamate	0.568577	0.032154	1.722924
Oxaziclomefone	0.559048	0.002161	1.135124
3,5-Dinitroguaiacol	0.554922	9.19E-05	1.265006
DL-2-Hydroxyvaleric acid	0.554918	0.000314	1.64695
Chrysoeriol 7-[feruloyl-(->2)-glucuronyl-(1->2)-glucuronide]	0.549963	6.13E-05	1.542608
Buprenorphine	0.544753	0.007559	4.315259
N-Arachidonoyl-L-Alanine	0.541462	0.001593	2.347112
2-Chloroethanol	0.535884	0.008633	2.178579
Isopentenyladenosine-5'-diphosphate	0.532122	0.002739	1.07771
Retinol	0.515577	1.11E-05	2.017928
[4-({[5,7-dihydroxy-2-(3,4,5-trihydroxyphenyl)-3,4-dihydro-2H-1-benzopyran-3-yl]oxy}carbonyl)-2,6-dihydroxyphenyl]oxidanesulfonic acid	0.514158	0.003108	1.030312
dTDP-4-amino-4,6-dideoxy-D-glucose	0.496785	0.000185	1.95969
Epigallocatechin 3-O-caffeate	0.496694	1.82E-05	2.203969
L-Cyclo	0.49155	0.00954	6.870637
PC(20:3(8Z,11Z,14Z)/0:0)	0.441903	0.000128	8.168778

3,3',5,5'-Tetrahydroxy-6,7-methyleneoxy-4'-methoxyflavone 3-glucuronide	0.434745	0.007685	1.694968
3-amino-octanoic acid	0.421982	0.024045	3.53711
LysoPE(20:3(5Z,8Z,11Z)/0:0)	0.420853	0.00968	1.322986
2-Chloroethanol	0.416959	0.04489	1.53908
{4-[2,3-dioxo-3-(2,4,6-trihydroxy-3-methoxyphenyl)propyl]-2-methoxyphenyl}oxidanesulfonic acid	0.416592	0.012978	1.470525
dTDP-L-mycarose	0.41644	0.000534	3.937428
N-Nitrosomethylvinylamine	0.403398	0.024054	2.087289
PC(7:0/O-8:0)	0.395338	0.004307	2.965882
2-Hydroxycinnamic acid	0.385631	0.003115	7.574149
PHENACYLAMINE	0.373892	0.005423	7.588013
gamma-Glutamylleucine	0.372912	0.036779	1.464854
PC(20:3(8Z,11Z,14Z)/18:2(9Z,12Z))	0.370141	0.038384	2.119952
Phosphatidylethanolamine lyso 16:0	0.363512	0.000184	1.772931
LysoPC(22:4(7Z,10Z,13Z,16Z))	0.362115	0.010827	1.955925
LysoPC(22:6(4Z,7Z,10Z,13Z,16Z,19Z))	0.357272	0.002558	1.579047
5,6-Dihydro-5-fluorouracil	0.354809	0.026697	1.056667
LysoPC(16:1(9Z))	0.350779	0.001615	5.468776
2-Chloroethanol	0.343463	0.029213	1.718119
3-Hydroxybenzaldehyde	0.33373	0.009327	4.450684
16alpha-Bromo-17beta-estradiol	0.329507	0.011375	2.481616
PC(18:4(9E,11E,13E,15E)/0:0)	0.321924	0.008139	1.013054
2-O-[2-O-(alpha-D-Mannopyranosyl)-alpha-D-glucopyranosyl]-3-phospho-D-glycerate	0.321299	0.009267	2.063932
(2-oxo-2,3-dihydro-1H-indol-3-yl)acetic acid	0.314399	0.000339	1.557748
Se-Methyl-L-selenocysteine	0.313306	0.018463	3.059098
PC(17:1(10Z)/0:0)	0.312569	0.011605	1.301949
NORLEUCINE	0.312443	0.016664	9.91791
PE(16:0/0:0)	0.31099	0.002794	3.507526
L-KYNURENINE	0.301232	0.000311	2.248077
3-Buten-1-amine	0.298976	0.006758	6.125716
1-(5'-Phosphoribosyl)-5-amino-4-(N-succinocarboxamide)-imidazole	0.289417	0.002078	1.643057
dTDP-3-methyl-4-oxo-2,6-dideoxy-L-allose	0.283475	0.029578	5.516213
3,4-Dihydro-6-methoxy-2,2-dimethyl-2H-1-benzopyran-4-ol	0.283108	0.049796	1.253719
(2R)-O-Phospho-3-sulfolactate	0.281074	0.046514	1.481207
2-Oxoglutaramate	0.278472	0.023604	3.729575
Isoleucine	0.275841	0.046503	11.0304
LysoPC(22:5(4Z,7Z,10Z,13Z,16Z))	0.271323	0.029309	2.519319
L-VALINE	0.262689	0.028397	5.255685
L-Valine	0.246517	0.021444	8.937189

PC(0:0/20:4(5Z,8Z,11Z,14Z))	0.237519	0.045948	8.841944
PE(18:0/0:0)	0.20191	0.016566	2.239399
Choline	0.187367	0.008078	7.887709
Tryptophan	0.175467	0.039368	4.5526
Idazoxan	0.166204	0.047709	7.647384
3-Nitroacrylate	0.153336	0.04227	1.337158
3-Nitroacrylate	0.145922	0.024895	2.03583
hydroxynefazodone	0.125167	0.014474	1.10382
Tambulin 3,5-diacetate	0.114064	0.000184	1.168525
Leucomycin V	-0.05162	0.019806	1.392308
L-GLUTAMINE	-0.09148	0.027943	3.552928
Pyroglutamic acid	-0.09887	0.023416	4.081818
GLYCERALDEHYDE	-0.13385	0.000693	3.966623
L-Selenocysteine	-0.14811	0.006586	4.795814
gamma-Glutamylglutamine	-0.16212	0.033364	2.139814
Imibencozole	-0.16873	0.010709	1.046652
C16 Sphinganine	-0.18324	0.035733	7.686699
Phloretin	-0.18532	0.033895	3.203772
Arsonoacetate	-0.20235	0.042942	1.296191
Novapikromyin	-0.21738	0.01522	1.198481
Glu-Gln	-0.23661	0.011597	1.088874
5-AMINOPENTANOATE	-0.26891	0.003364	15.6871
Janthitrem E	-0.27193	0.022584	1.196931
O-Toluidine	-0.28031	0.048885	2.388327
Melphalan	-0.37778	0.005282	1.255212
Dihydroxyaluminium	-0.38438	0.015978	2.67492
17-Octadecynoic Acid	-0.44498	0.024191	1.732163
Methyl methylthio selenide	-0.45203	0.045572	1.38806
PERILLIC ACID (-)	-0.47229	0.024986	2.627684
Diflunisal	-0.4916	0.00952	1.257416
Dioxoaminopyrine	-0.50941	0.006911	1.212984
Bowdichione	-0.5261	0.004824	2.756217
9E,11-Dodecadienal	-0.53326	0.025769	1.149813
Diflunisal	-0.5551	0.000159	3.132189
Demethylphosphinothricin	-0.56694	0.010882	1.011501
5,6-Dichloro-1,3-cyclohexadiene	-0.59835	0.000185	1.001822
Cysteineglutathione disulfide	-0.64141	0.005728	1.787779
DG(14:1(9Z)/17:2(9Z,12Z)/0:0)[iso2]	-0.66249	0.003448	1.410126
Endothion	-0.70721	0.028	4.70218
Palmyrolide A	-0.70838	0.041715	2.664176
Dihydrogriesenin	-0.77564	0.000497	1.247549
Adenosine	-0.81862	0.004163	1.684254

Abbreviations: VIP, variable importance in projection; FC, fold change.
